# Tracking COVID‐19 Severity and Progression Through Amines and Lipid Mediators

**DOI:** 10.1002/jmv.71030

**Published:** 2026-07-09

**Authors:** Melissa Goris, Merys Valdez, Lieke Lamont, Wei Yang, Amy Harms, Naama Karu, Sabine Bos, Jelte Geerlings, Tom Ottenhoff, Roula Tsonaka, Anna HE Roukens, M. Sesmu Arbous, Simone A. Joosten, Thomas Hankemeier, Alida Kindt, In collaboration with BEAT‐COVID group, M.S. Arbous, B.M. van den Berg, S. Cannegieter, C.M. Cobbaert, A. van der Does, J.J.M. van Dongen, J. Eikenboom, M.C.M. Feltkamp, A. Geluk, J.J. Goeman, M. Giera, T. Hankemeier, M.H.M. Heemskerk, P.S. Hiemstra, C.H. Hokke, J.J. Janse, S.P. Jochems, S.A. Joosten, M. Kikkert, L. Lamont, J. Manniën, T.H.M. Ottenhoff, M.R. del Prado, N. Queralt Rosinach, M. Roestenberg, M. Roos, A.H.E. Roukens, H.H. Smits, E.J. Snijder, F.J.T. Staal, L.A. Trouw, R. Tsonaka, A. Verhoeven, L.G. Visser, J.J.C. de Vries, D.J. van Westerloo, J. Wigbers, H.J. van der Wijk, R.C. van Wissen, M. Wuhrer, M. Yazdanbakhsh, M. Zlei

**Affiliations:** ^1^ Metabolomics and Analytics Center, Leiden Academic Center for Drug Research (LACDR) Leiden University Leiden The Netherlands; ^2^ Tasmanian Independent Metabolomics and Analytical Chemistry Solutions (TIMACS) Hobart Australia; ^3^ Leiden University Center for Infectious Diseases Leiden University Medical Center (LUMC) Leiden The Netherlands; ^4^ Department of Biomedical Data Sciences Leiden University Medical Center (LUMC) Leiden The Netherlands; ^5^ Department of Intensive Care Medicine Leiden University Medical Center (LUMC) Leiden The Netherlands; ^6^ Department of Internal Medicine Nephrology, The Leiden University Medical Center (LUMC) Leiden The Netherlands; ^7^ Department of Clinical Epidemiology The Leiden University Medical Center (LUMC) Leiden The Netherlands; ^8^ Department of Clinical Chemistry The Leiden University Medical Center (LUMC) Leiden The Netherlands; ^9^ Department of Pulmonary Medicine The Leiden University Medical Center (LUMC) Leiden The Netherlands; ^10^ Department of Immunology The Leiden University Medical Center (LUMC) Leiden The Netherlands; ^11^ Department of Internal Medicine Thrombosis and Hemostasis, The Leiden University Medical Center (LUMC) Leiden The Netherlands; ^12^ Center for Proteomics and Metabolomics The Leiden University Medical Center (LUMC) Leiden The Netherlands; ^13^ Department of Hematology The Leiden University Medical Center (LUMC) Leiden The Netherlands; ^14^ Department of Parasitology The Leiden University Medical Center (LUMC) Leiden The Netherlands; ^15^ Department of Human Genetics The Leiden University Medical Center (LUMC) Leiden The Netherlands

**Keywords:** corticosteroids, COVID‐19, immune markers, metabolomics, progression, severity

## Abstract

Coronavirus disease 2019 (COVID‐19) exhibits a broad spectrum of severity, ranging from mild to critical cases. To reduce mortality and costs, tailored early interventions for at‐risk patients are essential. This study elucidates the association between longitudinal metabolomic profiles and COVID‐19 severity and progression. We analyzed metabolomic profiles from a subset of the BEAT‐COVID cohort, a modestly sized yet well‐characterized and longitudinally sampled COVID‐19 cohort. The study comprises hospitalized patients sampled approximately twice weekly for up to 60 days after symptom onset during the first (29 patients, *n* = 92 samples) and second (48 patients, *n* = 112 samples) waves. Alongside sampling, daily disease severity was assessed using the Severity of Coronavirus Disease Assessment (SCODA) score. Associations were analyzed using univariate and multivariate linear mixed‐effect models. Perturbations in the tryptophan‐kynurenine pathway, linoleic acid derivatives, endocannabinoids, and sphingosines were significantly associated with disease severity and progression. These metabolic changes correlated with immune markers reflecting COVID‐19 severity. Some observed potential differences between the two waves could be partially attributed to the influence of corticosteroid treatment introduced during the second wave. Once confirmed in an independent validation cohort, the identified oxidative stress‐ and inflammation‐related metabolites may serve as biomarkers of disease progression, help identify at‐risk hospitalized patients, and provide potential therapeutic targets.

## 1 Introduction

Severe acute respiratory syndrome coronavirus 2 (SARS‐CoV‐2) caused a worldwide pandemic of coronavirus disease 2019 (COVID‐19), infecting over 777 million people and resulting in over 7 million reported deaths [1]. While the pandemic has ended, the virus remains endemic, with seasonal waves. COVID‐19 manifests with a range of severity, from mild symptoms to critical illness that requires invasive and costly interventions such as mechanical ventilation [1]. To reduce the severity and healthcare burden of COVID‐19, early and personalized interventions for at‐risk patients are essential. Identifying these patients at‐risk requires more precise markers than known risk factors, such as age, gender, obesity, and diabetes, which lack the specificity needed for personalized treatment [2]. Metabolomics can address this gap by capturing dynamic biochemical changes, enabling the discovery of biomarkers and pathways linked to disease progression.

Prior metabolomic studies used severity proxies such as intensive care unit (ICU) admission, survival, interleukin (IL)−6 levels, and World Health Organization (WHO) scores and revealed metabolic disruptions, including altered lipid and amino acid profiles [3, 4, 5, 6, 7, 8, 9]. For instance, patients admitted to the ICU showed varying levels of triglycerides, free fatty acids, oxylipins, and post‐translationally modified amino acids compared to those who could remain on the ward [5, 7]. Meanwhile, analyses of IL‐6 levels identified the involvement of the tryptophan‐kynurenine pathway, which plays a role in inflammation and immunity regulation [5, 7]. Additionally, alterations in circulating amino acids were associated with the WHO scale for COVID‐19 disease severity [6]. However, these proxies have limitations: the WHO scores and survival classification do not reflect daily fluctuations, while IL‐6 is a non‐specific marker for inflammation and is influenced by corticosteroid treatment introduced during the second wave [10]. Further, classifying patients' severity based on whether they stayed in the ward or required ICU care has become less reliable due to evolving care standards [11]. Moreover, ICU versus ward status may not purely reflect COVID‐19 severity, as admission is influenced by both disease severity and baseline metabolic or comorbidity status, potentially leading to spurious associations within the cohort. To overcome these limitations, the Severity of Coronavirus Disease Assessment (SCODA) score was utilized [12]. SCODA provides a continuous and objective measure of daily disease severity based on routine clinical data, including breathing and oxygenation parameters. The aim of this study is, therefore, to utilize the SCODA score to reveal novel insights with greater precision into the association between longitudinal metabolomic profiles and the severity and progression of COVID‐19.

This study investigates up to 80 immune markers and 207 metabolites in 77 patients hospitalized during the first two waves of the pandemic. The covered metabolic classes analyzed using mass‐spectrometry‐based profiling cover polyunsaturated fatty acids and their oxylipin derivatives, endocannabinoids, bile acids, (cyclic‐)lysophospholipids, and sphingolipids, as well as amino acids and their derivatives [5, 7]. In this study, the metabolic features will be (i) associated with SCODA‐based daily disease severity; (ii) associated with corticosteroid treatment; (iii) associated with the progression toward favorable or unfavorable outcomes; (iv) correlated with immune markers; and (v) integrated into multivariate molecular fingerprints associated with disease severity.

## 2 Methods

### 2.1 Study Cohort

The BEAT‐COVID study recruited patients with symptoms of COVID‐19 and a positive PCR test for SARS‐CoV‐2 admitted to Leiden University Medical Center (LUMC) in the Netherlands between April 2020 and March 2021 [12]. The patients were unvaccinated, ≥ 18 years old, and written informed consent was obtained from the patient or a representative. Patient care followed local and national guidelines, independent of study participation. The Leiden‐Den Haag‐Delft medical ethics committee provided ethical approval for this study protocol (NL73740.058.20), and the BEAT‐COVID study was registered in the Dutch Trial Registry (NL8589). One of the participants included here was excluded because their condition initially improved but later worsened after discontinuing corticosteroid treatment. Patients (*n* = 11) who were admitted to the hospital during the first wave but remained in the hospital during the second wave were only included with the data of the first wave. In addition, 24 samples from 13 patients and 7 samples from 5 patients from wave 1 and 2, respectively, were excluded as they exceeded 60 days since symptom onset. This resulted in a dataset of 77 patients (*n* = 204 samples). The patients were separated into two groups based on COVID‐19 waves and analyzed separately because of the change of care and availability of more sensitive mass spectrometers. The first COVID‐19 wave (May 2020) comprised 29 patients (*n* = 92 samples), and the second wave (June–December 2020) included 48 patients (*n* = 112 samples) (Supporting Information Table S1, S2).

### 2.2 Samples

EDTA blood plasma samples were collected by intravenous sampling three times a week during hospitalization and processed by the Department of Clinical Chemistry of the LUMC. The samples were stored at − 80°C until transportation, sub‐aliquoting, and metabolomic measurements. Along with the blood sampling, the Severity of Coronavirus Disease Assessment (SCODA) score was assessed, with the most critically ill patients being assigned the maximum score of 17 [12].

### 2.3 Metabolites

The metabolomic profiles of the plasma samples were obtained using mass spectrometry‐based metabolomics by the amines [5, 13] and signaling lipids [14] assays (Supporting Information Table S3, S4). Assay descriptions can be found in Supporting Information Tables S5 and S6, respectively. For both assays, quality control (QC) samples from pooled study samples were included to assess data quality by relative standard deviation of the QC samples [15]. In addition, blank samples (neat solution) were used to assess the background signal (Supporting Information Table S7) [15].

### 2.4 Immune Markers

The immune marker data were obtained as previously published (Supporting Information Table S8, S9) [16]. Briefly, four Bio‐Plex kits were used to measure cytokines and chemokines. Assays were performed according to the manufacturer's instructions with manufacturer‐specific standards and QC control samples. All samples were thawed and diluted 1:4 in sample Diluent HB and run as a single measurement with the streptavidin PE (1:200, Becton Dickinson, Erembodegem, Belgium) detection label. Samples were acquired on a Bio‐Plex 200 system and analyzed with Bio‐Plex manager software v6.2. Data of 105 analytes were received. For more details, see Supporting Information Table S10.

### 2.5 Statistical Analyses

All data pre‐processing and statistical analyses were performed in R (version 4.2.2). For a list of utilized packages, see Supporting Information Table S11. Data from the two COVID‐19 waves were separately analyzed. Briefly, metabolites and immune markers were excluded if they presented with fewer than three values per patient characteristic (Supporting Information Table S12). Seven additional and biologically relevant metabolite ratios were calculated (Supporting Information Table S13) [5, 17, 18, 19]. All these features were cube‐root transformed and auto‐scaled prior to statistical analyses. For the multivariate analyses, samples with data on both metabolites and immune markers are included. Metabolites and immune markers with more than 20% missing values were excluded, and the remaining missing values were imputed (see Supporting Information Table S14 for details). The associations between missing values and the factors of gender, ICU admission, and corticosteroid administration were assessed using Fisher's exact tests (Supporting Information Table S15). Univariate linear mixed effect (LME) models (Supporting Information Table S16 I–III) were calculated to identify the association of the metabolic profiles and i) SCODA score, ii) the impact of corticosteroid treatment, and iii) the progression of COVID‐19. These models were controlled for age, gender (f/m), ICU admission (y/n), and days since symptom onset, as well as for patient intervariability by including a random intercept per patient. A gender sensitivity analysis was performed using samples from the second wave. Multiple testing corrections were performed using the Benjamini‐Hochberg method and termed *q* values, where *q* < 0.05 was defined as significant, while *p* < 0.05 was considered a notable association. Direct *p* values were calculated by multiplying the sign of the estimate of the patient outcome with the ‐log10 *p* value. Pearson correlations for immune markers and metabolites with an association (*p* < 0.05) to the SCODA score were calculated. The multivariate metabolic signature defining the SCODA score was determined through stepwise feature selection optimizing the Akaike Information Criterion (AIC) for each wave (Supporting Information Table S17).

## 3 Results

### 3.1 Cohort Characteristics

The analyzed cohort comprises 77 patients sampled between May and December 2020. The two waves in this study were investigated separately due to changes in the protocol, not only for treatment but also for metabolomic analysis. This study investigated different outcomes, which required different data selections. Table 1, Supporting Information Tables S1 and S2 summarize the key characteristics of the patients in these datasets. The patients were separated by admission date and thus COVID‐19 waves to account for the change of care and available technology. The first COVID‐19 wave dataset (May 2020) comprised 29 patients (*n* = 92), and the second wave dataset (June–December 2020) included 48 patients (*n* = 112). See Supporting Information Table S13 for a detailed overview of the set of metabolites investigated in each analysis.

**Table 1:** Baseline patient characteristics of the COVID‐19 patients across the different analyses. Values are expressed as *n* (%) or median [full range]. The dataset containing all usable samples is utilized in the univariate analyses to assess the association with the SCODA score or corticosteroid treatment. The longitudinal data is used in the analyses to examine the association with the progression towards an unfavorable outcome. The imputed dataset is used for correlation and multivariate analyses. BMI: Body Mass Index; COPD: Chronic obstructive pulmonary disease; SCODA: severity of coronavirus disease assessment score.

	Full dataset		Only longitudinal profiles		Imputed dataset	
	Wave 1	Wave 2	Wave 1	Wave 2	Wave 1	Wave 2
Patients	29	48	26	20	28	27
Age	61 [18.5–77.5]	67 [33–88]	61 [30–77.5]	63 [33–77]	60.5 [18.5–77.5]	63 [33–88]
Men	23 (79.3%)	31 (64.6%)	22 (84.6%)	13 (65%)	23 (82.1%)	19 (70.4%)
BMI	29.1 [19.4– 36.2]	29.0 [17.6–46.9]	28.8 [19.4–33.6]	28.3 [17.6–41.4]	28.8 [19.4–36.2]	28.9 [17.6–41.4]
Diabetes	11 (37.9%)	18 (37.5%)	11 (42.3%)	5 (25%)	11 (39.3%)	10 (37%)
COPD	2 (6.9%)	3 (6.2%)	2 (7.7%)	1 (5%)	2 (7.1%)	3 (11.1%)
Days with symptoms until hospitalization	9 [0–21]	8.5 [0–34]	9 [0–21]	9 [0–15]	9 [0–21]	9 [0–34]
Days of hospitalization	39 [0–66]	8 [0–57]	41.5 [0–66]	12.5 [2–57]	39.5 [0–66]	14 [2–57]
Favorable outcome	—	—	15 (57.7%)	14 (70%)	—	—
Samples	92	112	89	73	90	81
Days since symptom onset	32.5 [7–57]	18 [4–50]	33 [7–57]	20 [10–50]	33 [7–57]	19 [4–50]
ICU	75 (81.5%)	53 (47.3%)	75 (84.3%)	40 (54.8%)	74 (82.2%)	45 (55.6%)
Treatment with antibiotics	58 (63%)	40 (35.7%)	57 (64%)	34 (46.6%)	58 (64.4%)	30 (37%)
Corticosteroid treatment	0 (0%)	70 (62.5%)	0 (0%)	37 (50.7%)	0 (0%)	43 (53.1%)
SCODA score	11 [0–17]	7 [0–17]	12 [0–17]	10 [0–17]	11.5 [0–17]	9 [0–17]
Thrombotic event	19 (20.7%)	8 (7.1%)	19 (21.3%)	4 (5.5%)	19 (21.1%)	3 (3.7%)

### 3.2 Metabolites Associate With the Severity of COVID‐19

**Figure 1:**
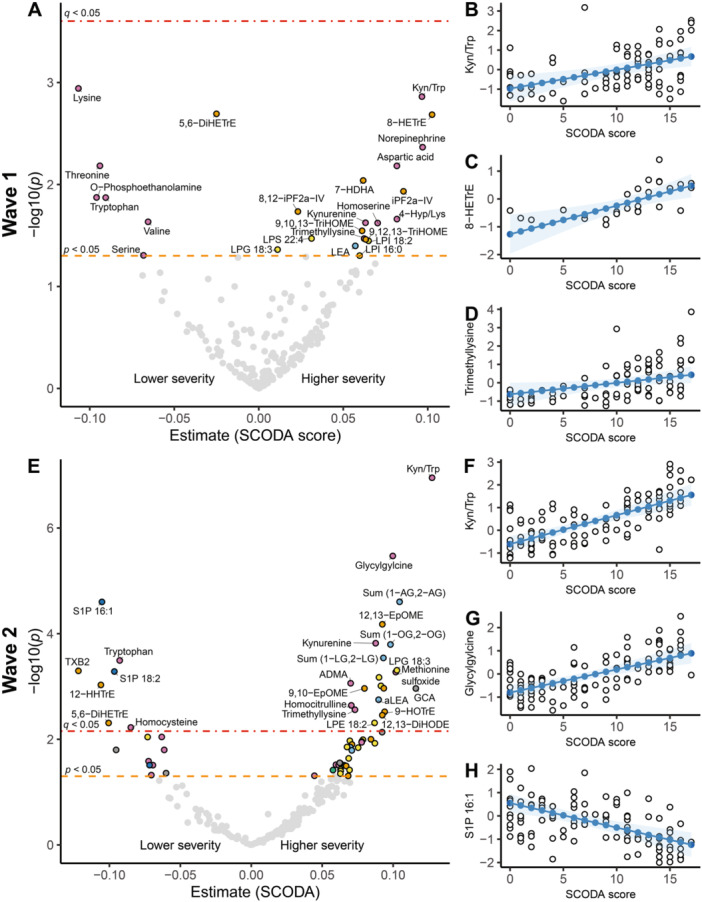
Univariate associations between metabolite level alterations and SCODA score. Volcano plots (A, E) show the longitudinal univariate associations between metabolite levels and SCODA scores using all samples from the first wave (*N* = 29 patients, 92 samples) and second wave (*N* = 48 patients, 112 samples), respectively. Each point represents a metabolite, with the x‐axis showing the model estimate and the y‐axis the –log10 *p* value. The metabolite points are colored according to their respective classes: amines (pink), bile acids (dark gray), eicosanoids (orange), oxylipins (light blue), free fatty acids (green), lysophospholipids (yellow), and sphingosines (dark blue). The horizontal orange dashed line marks the threshold for association (*p* < 0.05), while the red dot‐dash line indicates metabolites significantly associated after multiple testing correction (*q* < 0.05). Positive estimates reflect associations with an increase in SCODA score; negative estimates indicate associations with decreased scores. Effect plots (B–D for wave 1; F–H for wave 2) illustrate the relationship between SCODA score (range: 0–17) and metabolite levels. The blue line represents the LME model fit, with the light blue area indicating the 95% confidence interval (CI).

**Figure 2:**
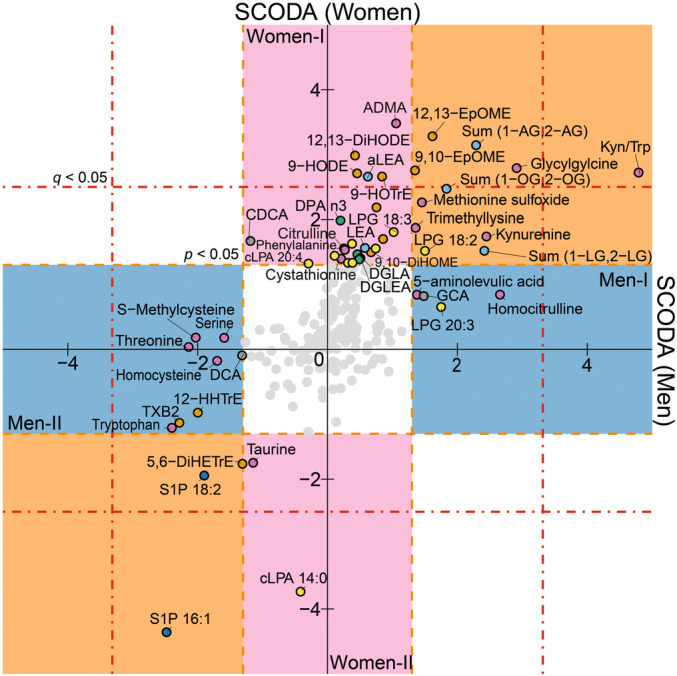
Gender‐stratified associations between metabolites and SCODA scores. The directed *p* plot compares the direction and significance of metabolite associations with SCODA scores between men (*N* = 31 patients, 79 samples) and women (*N* = 17 patients, 33 samples) from the second wave investigating all samples longitudinally. Directed *p* values are calculated by multiplying the sign of the model estimate by ‐log10 *p* value. Each dot represents a metabolite, colored according to its respective class: amines (pink), bile acids (dark gray), eicosanoids (orange), oxylipins (light blue), free fatty acids (green), lysophospholipids (yellow), and sphingosines (dark blue). Orange dashed lines indicate that metabolites plotted above the line are associated (*p* < 0.05), while red dot‐dash lines mark significance after multiple testing correction (*q* < 0.05). Metabolites within yellow squares showed an association in both men and women. Pink squares contain metabolites that showed association only in the analysis of the women population (Women‐I: positive associations; Women‐II: negative associations), and blue squares indicate metabolites associated solely in men (Men‐I: positive associations; Men‐II: negative associations).

The daily SCODA score is associated with metabolites measured on the same day in both wave 1 and wave 2 (Figure 1, Supporting Information Table S18). In wave 1, modest associations (*p* < 0.05) were observed between 25 metabolites and COVID‐19 severity. Of these 25 associations from wave 1, six metabolomic features showed significant associations (*q* < 0.05) with SCODA in wave 2, with the same direction of association (Supporting Information Figure S1). These features include the kynurenine‐to‐tryptophan ratio (Kyn/Trp), kynurenine, trimethyllysine, and lysophosphatidylglycerol (LPG) 18:3, associated with higher SCODA scores in both waves, along with 5,6‐dihydroxy‐eicosatrienoic acid (5,6‐DiHETrE) and tryptophan, which are associated with lower SCODA scores (Table 2). In wave 2, 28 out of 63 metabolomic features associated with the SCODA score (*p* < 0.05) passed multiple testing correction (*q *< 0.05, Figure 1E). Among these, 22 exhibited a positive association, including Kyn/Trp ratio (Figure 1F) and glycylglycine (Figure 1G), whereas six metabolites, such as sphingosine‐1‐phosphate (S1P) 16:1 (Figure 1H), showed a significant negative association. These named metabolites retained associations in the sensitivity analysis of the second wave stratified by gender (Figure 2: yellow squares, Supporting Information Figure S2). However, some associations noted in the main analysis were found exclusively in the men population, including the negative association with tryptophan (Figure 2: Men‐II). Additionally, men displayed a negative association between serine and SCODA, which was not observed in women or the overall population. Certain metabolites linked to SCODA in the main analysis were significantly associated exclusively in women, including asymmetric dimethylarginine (ADMA) (Figure 2: Women‐I) and the negatively associated cyclic‐lysophosphatidic acid (cLPA) 14:0 (Figure 2: Women‐II). Furthermore, additional metabolites demonstrated positive associations with SCODA solely within the women population, including omega‐3 docosapentaenoic acid (DPA n3), citrulline, and cystathionine.

**Table 2:** Overview of metabolomic features associated (*p* < 0.05) with SCODA in wave 1 and significantly linked (*q *< 0.05) in wave 2, maintaining the same association directionality. SE: standard error; *q*: FDR corrected *p* value.

	Wave 1				Wave 2			
	Model estimate	SE	*p*	*q*	Model estimate	SE	*p*	*q*
Kyn/Trp	9.66E‐02	2.92E‐02	1.37E‐03	1.03E‐01	1.27E‐01	2.24E‐02	1.12E‐07	2.31E‐05
kynurenine	6.32E‐02	2.74E‐02	2.37E‐02	2.98E‐01	8.77E‐02	2.23E‐02	1.52E‐04	4.74E‐03
tryptophan	−9.06E‐02	3.59E‐02	1.34E‐02	2.44E‐01	−9.27E‐02	2.49E‐02	3.21E‐04	7.39E‐03
LPG 18:3	1.11E‐02	5.43E‐03	4.35E‐02	3.78E‐01	1.03E‐01	2.84E‐02	4.93E‐04	8.49E‐03
trimethyllysine	6.24E‐02	2.89E‐02	3.39E‐02	3.40E‐01	7.31E‐02	2.38E‐02	2.76E‐03	2.48E‐02
5,6‐DiHETrE	−2.50E‐02	7.85E‐03	2.03E‐03	1.03E‐01	−1.01E‐01	3.49E‐02	4.90E‐03	3.76E‐02

**Figure 3:**
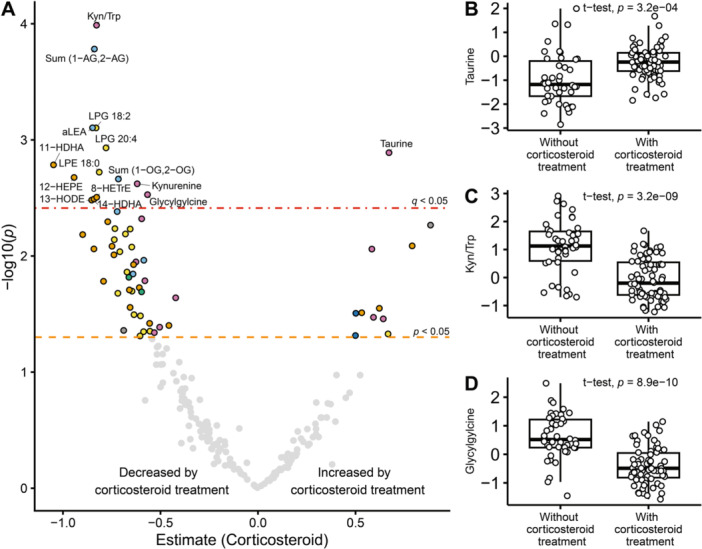
Impact of corticosteroid treatment on metabolite levels. The volcano plot (A) displays the longitudinal univariate associations between corticosteroid treatment and alterations in metabolite levels in patients using all second‐wave samples (*N* = 48 patients, 112 samples). Each point represents a metabolite, with the x‐axis showing the model estimate and the y axis the –log10 *p* value. Metabolites are colored by their respective class: amines (pink), bile acids (dark gray), eicosanoids (orange), oxylipins (light blue), free fatty acids (green), lysophospholipids (yellow), and sphingosines (dark blue). The horizontal orange dashed line marks the threshold for association (*p* < 0.05), while the red dot‐dash line indicates significance after multiple testing correction (*q* < 0.05). Positive estimates reflect associations between corticosteroid treatment and elevated metabolite levels; negative estimates indicate decreased levels. Boxplots (B–D) illustrate differences in metabolite levels between corticosteroid‐treated and untreated samples. Statistical significance of the boxplots is assessed using a t‐test.

Analysis of corticosteroid treatment in wave 2 (Figure 3, Supporting Information Table S19) demonstrated that corticosteroid use is significantly associated with an increase in taurine (Figure 3B) and a decrease in 14 metabolomic features, including Kyn/Trp ratio (Figure 3C) and glycylglycine (Figure 3D). Additionally, samples from patients receiving corticosteroid treatment showed levels of the microbial secondary bile acids deoxycholic acid (DCA) and its glycine conjugate glycodeoxycholic acid (GDCA) above the detection limit more frequently (Supporting Information Table S15). The similarities and differences between the impact of corticosteroid treatment on metabolites and their association with the SCODA score are visualized in a directed *p* plot (Supporting Information Figure S3). This plot reveals that eight metabolites positively associated with corticosteroid treatment are negatively associated with SCODA, including S1P 16:1. Conversely, 32 metabolomic features positively associated with SCODA exhibit a negative association with corticosteroid treatment, including Kyn/Trp ratio and the sum of arachidonoyl glycerols (AGs).

### 3.3 Metabolic Perturbations Associate With the Progression of COVID‐19

**Figure 4:**
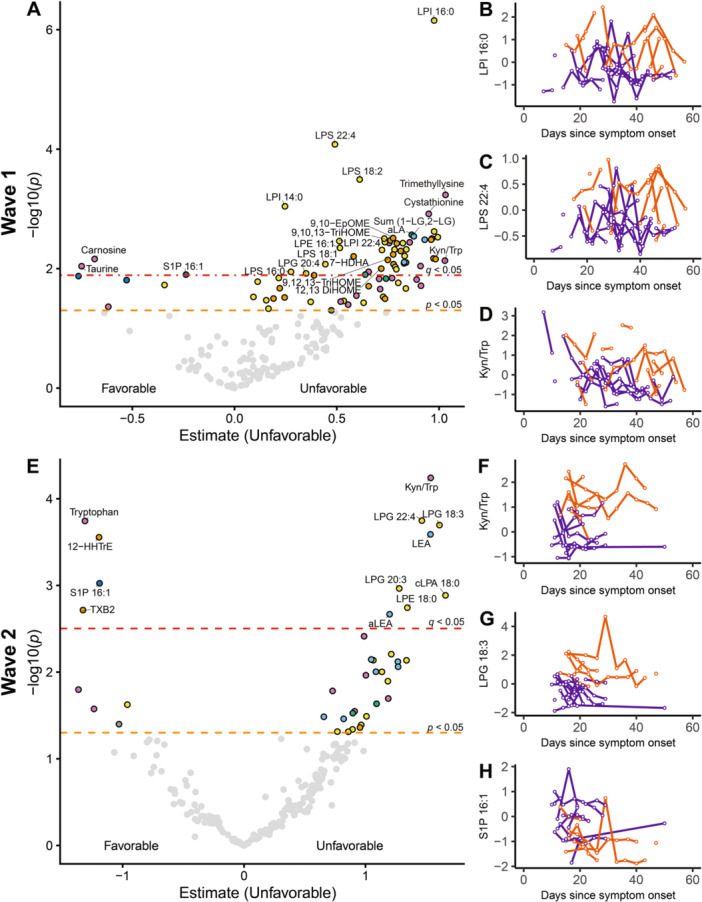
Univariate associations between longitudinal metabolite changes and COVID‐19 progression. The volcano plots (A, E) display ‐log10 *p* values plotted against the estimates of association with an unfavorable outcome for each metabolite. This analysis is based on the longitudinal profiles of patients using samples of all measured days from the first wave (*N* = 26 patients, 89 samples; Favorable outcome: *N* = 15 patients, 57 samples; Unfavorable outcome: *N* = 11 patients, 32 samples) and the second wave (*N* = 20 patients, 73 samples; Favorable outcome: *N* = 14 patients, 43 samples; Unfavorable outcome: *N* = 6 patients, 30 samples), respectively. Each point represents a metabolite, with the x‐axis showing the model estimate and the y‐axis the ‐log10 *p* value. The metabolites are colored by their respective class: amines (pink), bile acids (dark gray), eicosanoids (orange), oxylipins (light blue), free fatty acids (green), lysophospholipids (yellow), and sphingosines (dark blue). The horizontal orange dashed line marks the threshold for association (*p* < 0.05), while the red dot‐dash line indicates significance after multiple testing correction (*q* < 0.05). A positive estimate indicates an association of the metabolite with an unfavorable outcome, whereas a negative estimate indicates an association with a favorable outcome. Certain longitudinal metabolite profiles from the first wave (B–D) or the second wave (E–H) are plotted over time. Lines connect samples from the same patient, colored by patient outcome: purple for a favorable outcome and orange for an unfavorable outcome.

The health outcome of the patients was labeled as either favorable or unfavorable (Supporting Information Figure S4). A favorable outcome was assigned to patients who survived COVID‐19 during the study and had a final SCODA score ≤ 10. Patients who did not meet these criteria were classified as having an unfavorable outcome. Analyses of the longitudinal metabolomic data showed an association between metabolic perturbations and the progression towards an unfavorable outcome in both waves (Figure 4, Supporting Information Table S20). In the first wave, increased levels of 47 metabolomic features were significantly associated with progression to an unfavorable outcome (Figure 4A). These metabolites included 20 lysophospholipids like lysophosphatidylinositol (LPI)16:0 and lysophosphatidylserine (LPS) 22:4, and amines such as trimethyllysine and Kyn/Trp ratio (Figure 4B–C). In this analysis, only two metabolites were significantly associated with a favorable outcome, namely the amines taurine and carnosine (Figure 4D). The analysis of the second wave revealed that 12 metabolomic features were significantly associated with COVID‐19 progression (Figure 4E). An unfavorable outcome was significantly associated with increased levels of Kyn/Trp ratio (Figure 4F), five lysophospholipids including LPG 18:3 (Figure 4G) and the endocannabinoids linoleoyl ethanolamide (LEA) and alpha‐LEA (aLEA). Patients with a favorable outcome showed significantly increased levels of tryptophan, S1P 16:1 (Figure 4H), 12‐hydroxy‐heptadecatrienoic acid (12‐HHTrE) and thromboxane B2 (TXB2).

### 3.4 Metabolites Correlate With Immune Markers Reflecting COVID‐19 Severity

**Figure 5:**
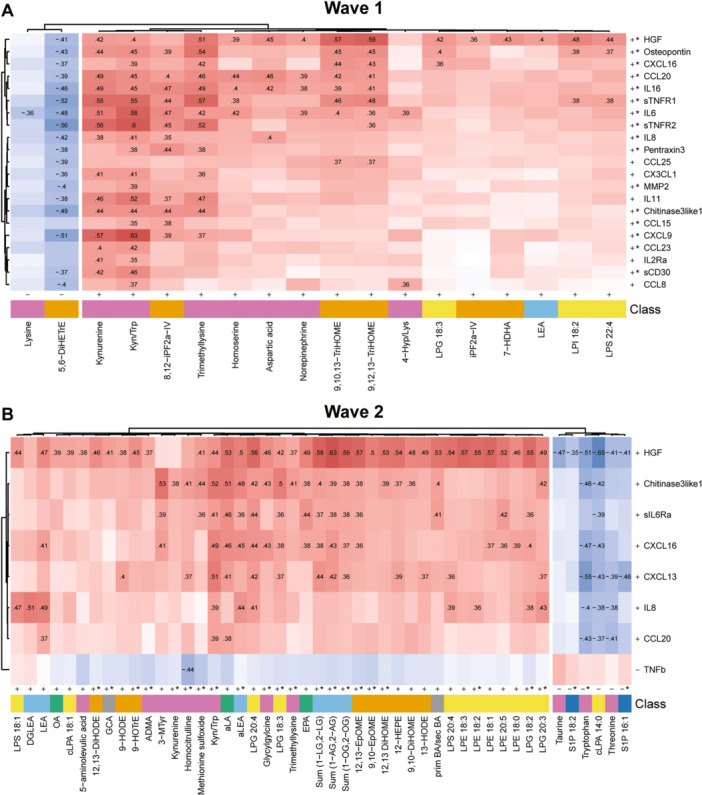
Correlation of metabolites with immune markers. Hierarchical clustering heatmaps show the Pearson correlation between immune markers and metabolites using all day samples of patients in the first wave (A; *N* = 28 patients, 90 samples) and second wave (B; *N* = 27 patients, 81 samples). The plots include only metabolites and immune markers that were associated with SCODA (*p* < 0.05) in the SCODA analysis. This association with the SCODA score is indicated in the annotation rows, with a “+” for a positive association, a “–” for a negative association, and a “*” for an FDR corrected significant association. The colored boxes indicate the value of the Pearson correlation (dark blue –1 to dark red 1), which is specified when it exceeds |0.35|. Only metabolites and immune markers with at least one correlation greater than |0.35| are shown in the heatmap. The metabolite classes are annotated in the class row, colored according to their respective classes: amines (pink), bile acids (dark gray), eicosanoids (orange), oxylipins (light blue), free fatty acids (green), lysophospholipids (yellow), and sphingosines (dark blue).

Pearson correlation analyses showed that the immune markers supported the trend observed for the metabolites (Figure 5, Supporting Information Figure S5). Metabolites positively associated with SCODA were also positively correlated with SCODA‐associated immune markers, such as hepatocyte growth factor (HGF), IL‐6, and chemokine (C‐X‐C motif) ligand (CXCL)−16. In a similar manner, metabolites negatively associated with SCODA displayed negative correlations with these immune markers. In the first wave, correlations were more apparent for metabolomic features that exhibited a notable association with COVID‐19 across multiple analyses, such as Kyn/Trp ratio and the negatively associated metabolite 5,6‐DiHETrE (Figure 5A, Supporting Information Table S21). In the second wave, similar correlations were observed (Figure 5B, Supporting Information Table S22). For instance, HGF is linked to SCODA and demonstrated a positive correlation with most metabolomic features associated with increased SCODA, including Kyn/Trp ratio, alpha‐linolenic acid (aLA), and the sums of AGs, linoleoyl glycerols (LGs), and oleoyl glycerol (OGs). Additionally, HGF showed a negative correlation with metabolites such as tryptophan, taurine, S1P 16:1, and S1P 18:2, which were negatively associated with SCODA.

### 3.5 Multivariate Fingerprint for Disease Severity

To determine which combinations of biological factors best explain disease severity, statistical models incorporating metabolites and/or immune markers are developed and evaluated (Table 3). The models are optimized to effectively capture essential patterns while avoiding overfitting. During the first wave, the identified signature included hospitalization status labeled ICU and 30 metabolomic features, amongst which were, for example, Kyn/Trp ratio, 8,12‐iso‐Prostaglandin F2a‐IV (8,12‐iPF2a‐IV), and LPG 18:3, that were also seen in other analyses. In the second wave, the optimized model comprised hospitalization status and 17 metabolomic features, including the Kyn/Trp ratio, S1P 16:1, and 8‐HETrE.

**Table 3:** Multivariable models built using distinct null models and combinations of addable features. The addable features include (a combination of) metabolites (wave 1: K = 185; wave 2: K = 200), immune markers (wave 1: K = 64; wave 2: K = 60), and the confounders age, gender, ICU admission, days since symptom onset, and corticosteroid treatment (wave 1: K = 4; wave 2: K = 5; corticosteroid treatment included in wave 2 only). Models are optimized to minimize Akaike Information Criterion (AIC), balancing fit and complexity.

AIC	Null model	Type of addable variables		Added variables
A) Wave 1				
AIC				
334.8	Random intercept per patient	Metabolites Confounders		ICU, Kyn/Trp, LPG 22:4, Lysine, LPS 18:2, DGLA, 7‐HDHA, cLPA 18:0, LPI 22:4, LPG 14:0, LPG 18:3, aLEA, LPG 20:5, 8,12‐iPF2a‐IV, 20‐HETE, O‐Phosphoethanolamine, Glycylgylcine, LPG 22:6, LPG 16:1, LPG 20:3, LPG 18:1, LPI 14:0, TLCA_3S, GLCA, 8,9‐DiHETrE, cLPA 20:4, LPI 22:6, LPG 20:4, Homoserine, LPE 14:0, Kynurenine
350.4	Random intercept per patient Confounders	Metabolites		Kyn/Trp, LPG 22:4, Lysine, LPS 18:2, LPG 14:0, 20‐HETE, LPG 18:3, DGLA, LPI 22:4, cLPA 18:0, 7‐HDHA, aLEA, 8,12‐iPF2a‐IV, LPG 20:5, O‐Phosphoethanolamine, Glycylgylcine, LPG 22:6, LPG 16:1, LPG 18:1, LPG 20:3, TLCA_3S, GLCA, LPI 14:0, cLPA 20:4, LPG 20:4, LPI 22:6, 8,9‐DiHETrE, Homoserine, LPE 14:0
367.5	Random intercept per patient Confounders	Metabolites Immune markers		CCL20, CCL15, O‐Phosphoethanolamine, LPG 22:4, Glycylgylcine, Valine, LPG 14:0, LPI 16:1, 20‐HETE, LPS 18:2, IL11, Homoserine, APRIL, sTNFR2, LPI 16:0, LPI 14:0, LPG 16:1, LPG 20:5
369.9	Random intercept per patient	Metabolites Immune markers Confounders		ICU, CCL20, CCL15, LPG 22:4, Kyn/Trp, LPA 22:4, LPG 14:0, LPI 16:0, DGLA, cLPA 16:1, O‐Phosphoethanolamine, LPI 22:4, 20‐HETE, Glycylgylcine, Valine, LPI 14:0, LPA 14:0
424.5	Random intercept per patient	Immune markers Confounders		ICU, CCL20, CCL15, CCL23
428.7	Random intercept per patient Confounders	Immune markers		CCL20, CCL15, IL11, CCL27, CCL23
B) Wave 2				
AIC				
303.1	Random intercept per patient	Metabolites Confounders	ICU, Glycylgylcine, Kyn/Trp, 5‐hydroxy trypophan, 3‐MTyr, Homocitrulline, S1P 16:1, LPG 18:3, CDCA, 15‐HEPE, O‐Acetylserine, Putrescine, LPG 14:0, Valine, LPI 14:0, 8‐HETrE, LPG 20:3, Trimethyllysine	
313.5	Random intercept per patient	Metabolites Immune markers Confounders	ICU, Glycylgylcine, Kyn/Trp, 5‐hydroxy trypophan, 3‐MTyr, Homocitrulline, Phenylalanine, S1P 16:1, LPG 18:3, CDCA, 15‐HEPE, O‐Acetylserine, sTNFR1, CCL26	
337.3	Random intercept per patient Confounders	Metabolites	Glycylgylcine, Kyn/Trp, Homocysteine, TLCA_3S, S1P 16:1, LPG 20:3, Proline, Valine, CDCA	
337.3	Random intercept per patient Confounders	Metabolites, Immune markers	Glycylgylcine, Kyn/Trp, Homocysteine, TLCA_3S, S1P 16:1, LPG 20:3, Proline, Valine, CDCA	
392.8	Random intercept per patient	Immune markers Confounders	ICU, HGF	
397.0	Random intercept per patient Confounders	Immune markers	HGF, Pentraxin3	

## 4 Discussion

This study aimed to identify metabolomic proxies of COVID‐19 disease severity and progression. To this end, the daily Severity of Coronavirus Disease Assessment (SCODA) score was utilized to investigate associations with longitudinal metabolomic profiles. All metabolites were measured in plasma, which integrates biochemical signals from multiple tissues rather than just the lungs or other specific organs. Therefore, these signals show overall host responses to disease severity. Some modest but noticeable differences were observed between the first and second waves, with certain distinct metabolites also associated with the corticosteroid treatment introduced in the second wave. The metabolomics results correlated with immune markers reflecting COVID‐19 severity. Lastly, multivariate models were established to describe the daily COVID‐19 severity.

Our study showed that COVID‐19 severity and progression towards unfavorable outcomes are associated with elevated kynurenine levels, reduced tryptophan levels, and an increased Kyn/Trp ratio. These results support previous research linking a high Kyn/Trp ratio to severe COVID‐19 cases, ICU admissions, lower survival rates, and elevated IL‐6 [5, 9, 20, 21, 22]. Tryptophan is metabolized into kynurenine via the kynurenine pathway, which is enzymatically upregulated in response to inflammatory factors such as TNF‐α and IL‐6 [23]. Our study also observed medium‐to‐strong correlations between the Kyn/Trp ratio and pro‐inflammatory markers, including CXCL16, IL‐6, and soluble TNF receptors [23]. Kynurenine acts as a pro‐inflammatory mediator, further exacerbating the inflammatory response. This accumulation of kynurenine is linked to various conditions including hyperinflammation syndrome, a key factor in COVID‐19 severity and mortality [24, 25].

During the second COVID‐19 wave, the WHO recommended corticosteroid treatment for hospitalized patients with severe cases, leading to routine administration during hospitalization [10]. It has been demonstrated that corticosteroid treatment alters the metabolomic profile of COVID‐19 patients [26, 27]. Our analysis revealed additional negative associations between corticosteroid treatment and most metabolites linked to high disease severity, including Kyn/Trp ratio (Supporting Information Figure S3). Corticosteroid treatment was also associated with decreased levels of the post‐translational modification products glycylglycine and 1‐methylhistidine, which exhibited distinct trends between the waves. The lower post‐translational modification products may reflect a reduction in oxidative stress burden and in the innate inflammatory response, stemming from the anti‐inflammatory effects of corticosteroids [28]. Furthermore, 1‐methylhistidine, which can originate in muscle tissue breakdown, has also been identified as a potential biomarker for drug‐induced skeletal muscle toxicity, one of the side effects of corticosteroid treatment [28, 29].

Our study associated the severity of COVID‐19 with alterations in fatty acid and oxylipin levels, specifically elevated levels of linoleic acid (LA) and its derivatives. Enzymatic (cytochrome P450 (CYP) and soluble epoxide hydrolase (sEH)) and non‐enzymatic peroxidation derivatives of LA show a significant positive association with an unfavorable outcome in our findings, expressed by increased dihydroxy‐octadecenoic acids (DiHOMEs) and epoxyoctadecenoic acids (EpOMEs) levels. The activation role of EpOMEs was also observed in respiratory burst, an immune cell response in which phagocytes rapidly increase the production of reactive oxygen species (ROS) during phagocytosis [30]. The EpOMEs and DiHOMEs play a role in the inflammatory response triggered by environmental insults in the lungs [31, 32]. In addition, 9,10‐DiHOME, known as ‘leukotoxin‐diol’, was associated with acute respiratory distress syndrome (ARDS), which is a symptom of advancing COVID‐19 [24, 33]. These findings are in line with a previous study that demonstrated an association between elevated levels of LA and some of its derivatives and severe inflammation in patients admitted to the ICU with COVID‐19 [7].

LA is converted into arachidonic acid (AA), a precursor of leukotrienes and prostanoids as well as other oxylipins (5‐HETE etc.). The upregulation of the non‐enzymatic peroxidation products of AA, namely the isoprostanes iPF2a‐IV and 8,12‐iPF2a‐IV, with higher SCODA scores, confirms the increased oxidative stress which accompanies the inflammatory response in more severe COVID‐19 patients. Both 12‐HHTrE and TXB2 are AA products of thromboxane A2 synthase (TXA2) activity, which is responsible for vascular function and platelet aggregation [34]. However, despite the instability of TXA2, 12‐HHTrE levels are still found in high abundance, which suggests non‐enzymatic oxidation of AA [34, 35]. Dalli et al. associated pro‐inflammatory mediators (such as TXB2) and pro‐resolving mediators with the development of ARDS in sepsis patients; sepsis is observed in severely ill patients with COVID‐19, particularly younger adults [36, 37, 38]. Interestingly, in their results, TXB2 was linked to sepsis survival, a finding similar to our observation, in which TXB2 was associated with lower SCODA scores and a favorable outcome in wave 2. As a result, it can be anticipated that lower platelet‐derived TXB2 levels may impede the shift in macrophage polarization (from pro‐inflammatory to anti‐inflammatory) [39].

Omega‐3 fatty acids, eicosapentaenoic acid (EPA) and docosahexaenoic acid (DHA), are the precursors of anti‐inflammatory modulators, such as resolvins, protectins and maresins. Hydroxy‐DHAs (HDHA), formed via LOX activity, were downregulated with corticosteroid treatment and associated with an unfavorable outcome in wave 1 [40]. In addition, multiple HDHA metabolites (X‐HDHA) correlated positively with the macrophage‐activation marker sCD163 (Supporting Information Table S22). Although they are the precursors of oxylipins involved in the resolution of inflammation, our results did not reflect such effect for 7‐HDHA, 11‐HDHA, and 14‐HDHA. This has been observed in another COVID study [7] and may also suggest an increased production (perhaps to address the inflammatory burden), or merely peroxidation of DHA, without activation of the enzymes that enable the next steps in the production of resolvins, protectins, and maresins.

Our results indicate that elevated endocannabinoid levels are associated with the severity of COVID‐19, aligning with another study conducted during the first COVID‐19 wave, which showed higher levels in ICU patients compared to ward patients, particularly AGs, LGs, and OGs [7]. Furthermore, our analysis demonstrated that these endocannabinoids were also significantly associated with the progression towards an unfavorable outcome. The examination of the second wave revealed additional associations with endocannabinoids, including LEA, aLEA, dihomo‐gamma‐LEA (DGLEA), docosahexaenoyl ethanolamide (DHEA), and N‐arachidonoylethanolamine (AEA). Beyond fatty acid breakdown products and central nervous system modulators and reserves of precursors for fatty acids and oxylipins, they are also central nervous system modulators and function as regulators in various immune cells [41]. Therefore, modulation of the endocannabinoid system appears to be part of the immune response, where its dysregulation may contribute to hyperinflammation and prolonged inflammatory signaling [41].

In contrast to most other lipids, elevated levels of sphingosine‐1‐phosphates (S1P) were associated with reduced COVID‐19 severity and favorable disease progression in both waves. This observation aligns with studies examining S1P levels in patients with COVID‐19 in ICU versus ward, supporting the protective role of S1P in modulating immune responses and maintaining vascular integrity [42, 43]. S1Ps aid in resolving inflammation, for instance, through their release by alveolar macrophages during acute lung injury and have also been shown to serve as a target to mitigate central inflammation in COVID‐19 [44, 45, 46]. By activating endothelial S1P receptors, S1P can function as a vasodilator, thereby preserving endothelial integrity and maintaining lung permeability. Consistent with this protective role, corticosteroid treatment was positively associated with S1Ps in our cohort. Corticosteroids can induce sphingosine kinase 1, elevating circulating S1P and thereby contributing to endothelial protection and resolution of lung inflammation [27, 47]. Taken together, our findings support the hypothesis of S1P analogs as a potential treatment for COVID‐19 patients.

Our study highlights notable gender‐specific differences in the relationship between metabolites and COVID‐19 severity, as previously suggested [48]. Some associations, such as ADMA, appear to be driven primarily by women. ADMA, a post‐translational modification product of arginine residues in proteins, increases during oxidative stress and inflammation. Its levels have been associated with COVID‐19 severity and mortality, either as a reporting marker or possibly due to its active inhibition of nitric oxide synthase, which is essential for endothelial function and modulation of nitrosative stress [5, 49]. Although previous research indicates no significant gender‐related differences in ADMA as a cardiovascular risk factor, the influence of sex hormones, particularly estrogen in women, suggests better protection from oxidative stress and inflammation, including their effects on endothelial and mitochondrial function [50, 51].

While DPA n3 and citrulline are generally associated with milder COVID‐19 outcomes, we observed an association between these metabolites and higher SCODA scores in women [50, 51]. This paradox may reflect a more robust immune response in women, as previously hypothesized [48]. This hypothesis is further supported by our finding that elevated cystathionine levels were associated with SCODA scores exclusively in women. This finding mirrors earlier observations from a first‐wave cohort and suggests a potential role for estrogen, which is known to regulate cellular cystathionine levels [5, 52]. Altogether, our findings support gender‐related differences in metabolic profiles associated with COVID‐19 severity. However, it is important to acknowledge the limited and imbalanced sample size of women patients, which also hindered sensitivity analyses of the first wave. Therefore, future studies are required to further assess gender‐specific differences in this context.

This study has certain limitations to consider. First, it examined metabolite associations with COVID‐19 severity, which precludes drawing causal conclusions. Second, circulating metabolites in the plasma samples reflect systemic metabolism and may not directly mirror intracellular, lung‐specific or tissue‐specific biochemical activity. Third, the study design relied on available patient samples, leading to imbalanced cohorts and differences in hospital admission criteria between waves. During the first wave, only the most severe cases were hospitalized and typically admitted later in the disease course, whereas in the second wave, widespread test availability enabled earlier diagnosis (Supporting Information Figure S6). Furthermore, while the new mass spectrometer allowed for more metabolites to be investigated in the second wave, a direct comparison between waves was not possible. To address this limitation, the samples were analyzed separately by wave. Fourth, this study focused on hospitalized patients, providing detailed insights into severe cases, though findings may not fully extend to milder non‐hospitalized cases. Fifth, detailed medication data were not consistently available. While corticosteroid treatment timing was recorded, the use of other medications, including antibiotics, was not systematically captured and could therefore not be adequately accounted for. Lastly, the limited sample size hindered the investigation of rare cases such as corticosteroid treatment failure. Further metabolomic studies of these cases may provide valuable insights into corticosteroid treatment resistance to inform personalized therapies.

In conclusion, this study identified perturbations in amines and signaling lipids associated with the severity and progression of COVID‐19. These perturbations were primarily related to oxidative stress and the modulation of the inflammatory response. This study supports the well‐described upregulation of the tryptophan–kynurenine pathway in COVID‐19 patients and demonstrates an association with disease severity and progression. Endocannabinoids, oxylipins, and lysophospholipids are broadly elevated in systemic illnesses marked by inflammation, oxidative stress, and tissue damage [53]. Thus, the observed associations between these lipid mediators and COVID‐19 severity likely reflect a general disease signature rather than COVID‐19–specific metabolic alterations. Once confirmed in an independent validation cohort, the altered metabolites serve as potential biomarkers for monitoring disease severity and identifying at‐risk hospitalized patients. Furthermore, they support the potential use of modulators of the tryptophan‐kynurenine pathway, endocannabinoid system, and sphingosine‐1‐phosphate analogs as therapeutic targets for the treatment of COVID‐19.

## In Collaboration With the BEAT‐COVID Group

M.S. Arbous^1^, B.M. van den Berg^2^, S. Cannegieter^3^, C.M. Cobbaert^4^, A. van der Does^5^, J.J.M. van Dongen^6^, J. Eikenboom^7^, M.C.M. Feltkamp^8^, A. Geluk^8^, J.J. Goeman^9^, M. Giera^10^, T. Hankemeier^11^, M.H.M. Heemskerk^12^, P.S. Hiemstra^5^, C.H. Hokke^13^, J.J. Janse^13^, S.P. Jochems^13^, S.A. Joosten^8^, M. Kikkert^8^, L. Lamont^11^, J. Manniën^9^, T.H.M. Ottenhoff^8^, M.R. del Prado^1^, N. Queralt Rosinach^14^, M. Roestenberg^13^, M. Roos^14^, A.H.E. Roukens^8^, H.H. Smits^13^, E.J. Snijder^8^, F.J.T. Staal^6^, L.A. Trouw^6^, R. Tsonaka^9^, A. Verhoeven^10^, L.G. Visser^8^, J.J.C. de Vries^8^, D.J. van Westerloo^1^, J. Wigbers^1^, H.J. van der Wijk^9^, R.C. van Wissen^4^, M. Wuhrer^10^, M. Yazdanbakhsh^13^, M. Zlei^6^

1. Dept. of Intensive Care, LUMC, Leiden, The Netherlands

2. Dept. of Internal Medicine, Nephrology, LUMC, Leiden, The Netherlands

3. Dept. of Clinical Epidemiology, LUMC, Leiden, The Netherlands

4. Dept. of Clinical Chemistry, LUMC, Leiden, The Netherlands

5. Dept. of Pulmonary Medicine, LUMC, Leiden, The Netherlands

6. Dept. of Immunology, LUMC, Leiden, The Netherlands

7. Dept. of Internal Medicine, Thrombosis and Hemostasis, LUMC, Leiden, The Netherlands

8. Dept. of Infectious Diseases, LUMC, Leiden, The Netherlands

9. Dept. of Biomedical Data Sciences, LUMC, Leiden, The Netherlands

10. Center for Proteomics and Metabolomics, LUMC, Leiden, The Netherlands

11. Metabolomics and Analytics Centre, Leiden Academic Center for Drug Research, Leiden University, Leiden, The Netherlands

12. Dept. of Hematology, LUMC, Leiden, The Netherlands

13. Dept. of Parasitology, LUMC, Leiden, The Netherlands

14. Dept. of Human Genetics, LUMC, Leiden, The Netherlands

## Consent

Written informed consent was obtained from the patient or a representative.

## Conflicts of Interest

The authors declare that they have no known competing financial interests or personal relationships that could have appeared to influence the work reported in this paper.

## Supporting information

Supporting File S1

Supporting File S2

## Data Availability

The raw data are provided in the Supporting Materials, and additional data are available upon reasonable request. The data that supports the findings of this study are available in the supporting material of this article.
