# Supplementary material for: Tracking COVID‐19 Severity and Progression Through Amines and Lipid Mediators
Source: J Med Virol. 2026 Jul 9;98(7):e71030. doi: 10.1002/jmv.71030 (PMC13347278; doi:10.1002/jmv.71030)
Supplement: Supplementary file 1 — Supporting File S1 [file JMV-98-e71030-s002.docx]

**
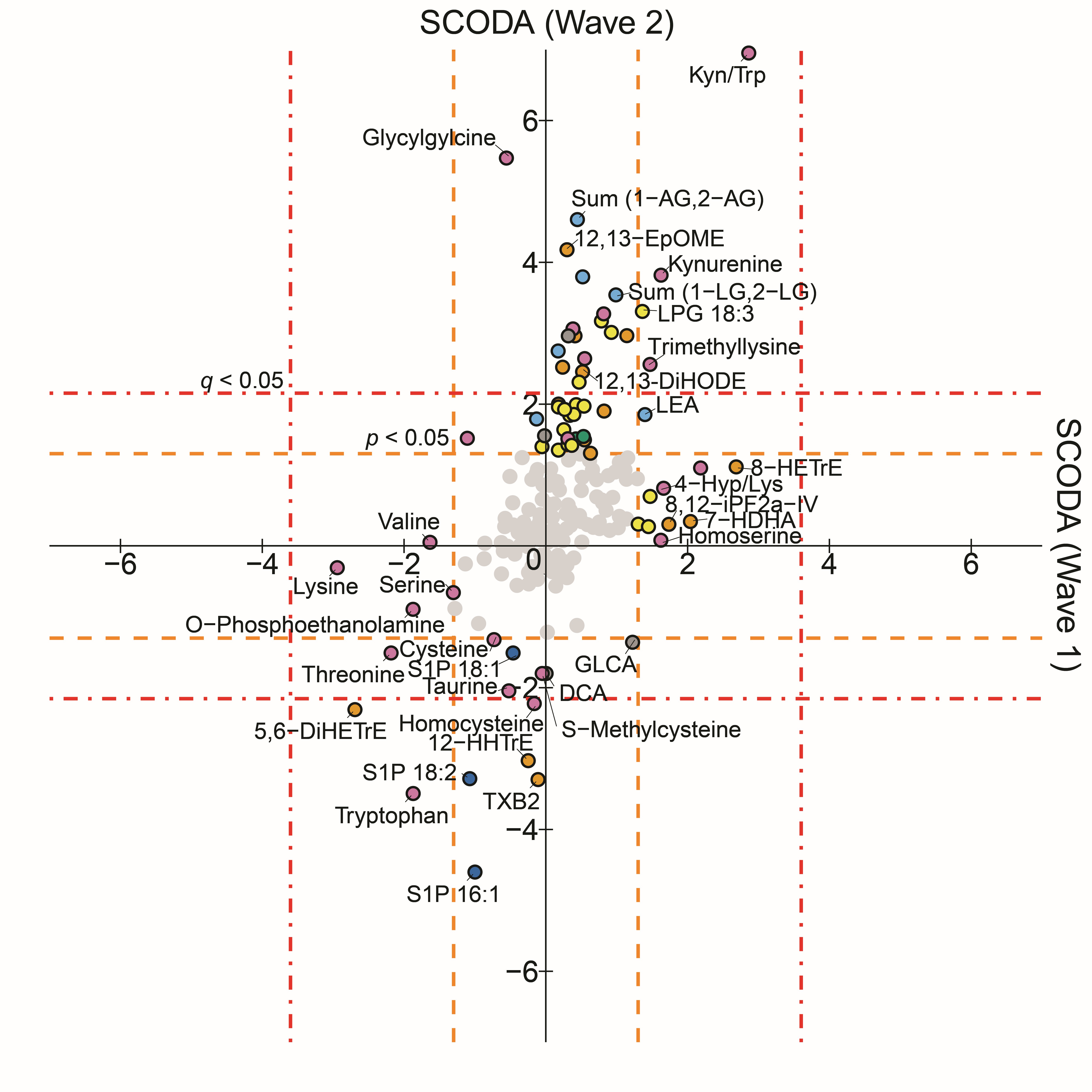
Figure S1. Comparison of metabolites-SCODA associations between the waves.** The directed *p* plot compares the direction and significance of the association between metabolites and the SCODA score in the first and second waves. The directed *p* value is calculated by multiplying the sign of the estimate by the -log10 *p* value. The directed *p* values from both waves are plotted against one another. The dots represent metabolites, colored according to their respective classes: amines (pink), bile acids (dark gray), eicosanoids (orange), oxylipins (light blue), free fatty acids (green), lysophospholipids (yellow), and sphingosines (dark blue). The orange dashed lines indicate that metabolites plotted above the line are associated (*p* < 0.05) with the SCODA score. The red dot-dash lines signify that metabolites above this line are significantly (*q* < 0.05) associated with the SCODA score.

**
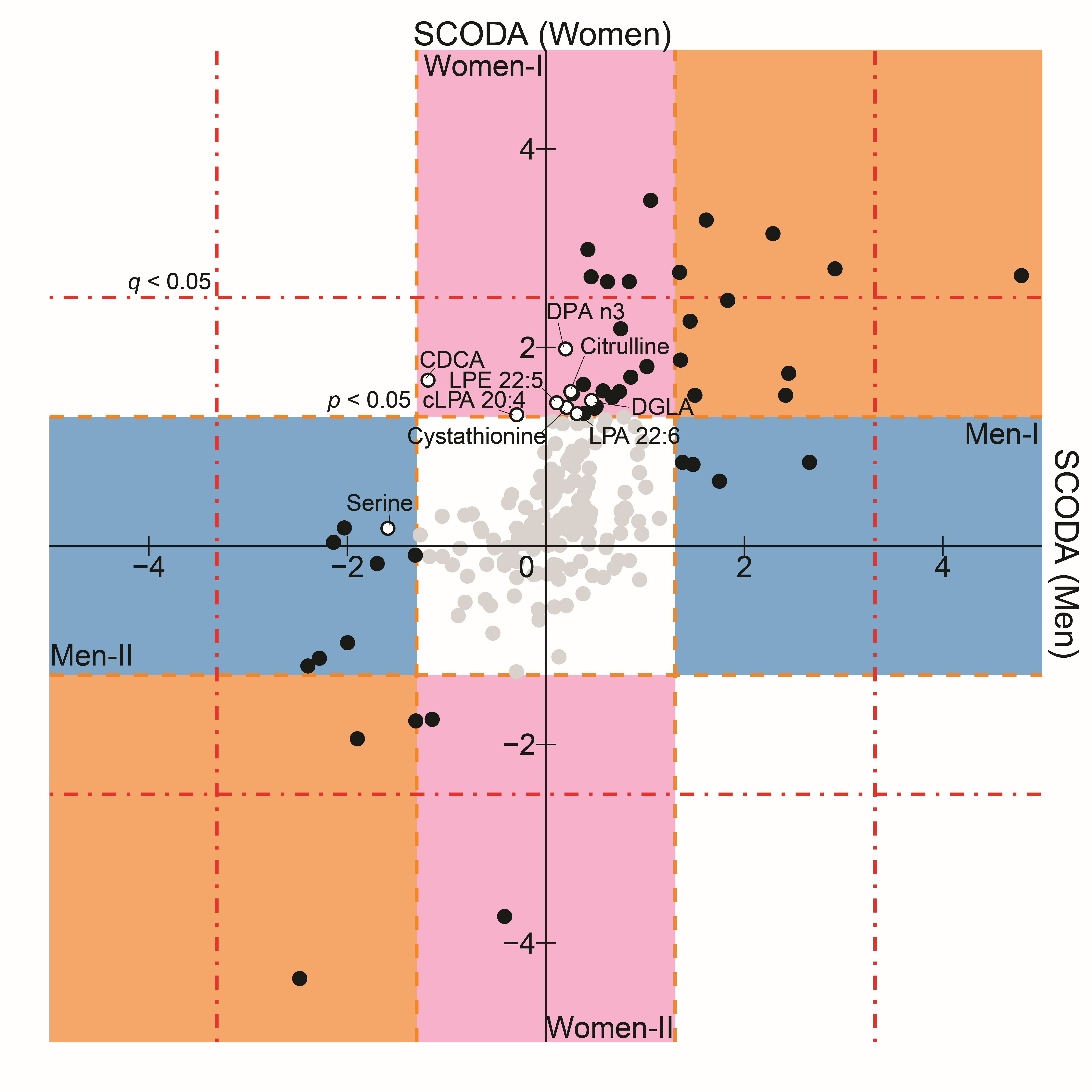
Figure S2. Gender-stratified associations between metabolites and SCODA scores.** The directed *p* plot compares the direction and significance of metabolite associations with SCODA scores between men (*N* = 31) and women (*N* = 17) from the second wave. Directed *p* values are calculated by multiplying the sign of the model estimate by -log10 *p* value. Each dot represents a metabolite, colored according to their association with SCODA in the full analysis: associated *p* < 0.05 (black) or not associated *p* > 0.05 (white). Orange dashed lines indicate that metabolites plotted above the line are associated (*p* < 0.05), while red dot-dash lines mark significance after multiple testing correction (*q* < 0.05). Metabolites within yellow squares showed an association in both men and women. Pink squares contain metabolites that showed association only in the analysis of the women population (Women-I: positive associations; Women-II: negative associations), and blue squares indicate metabolites associated solely in men (Men-I: positive associations; Men-II: negative associations).

**

**

**Figure S3**. **Directed *p* plot of the association of metabolite level alterations with corticosteroid treatment and SCODA score.** The directed *p* plot compares the direction and significance of corticosteroid treatment-associated changes in metabolite levels with the association between metabolites and the SCODA score. The directed *p* value is calculated by multiplying the sign of the estimate by the -log10 *p* value. The directed *p* values from the corticosteroid analysis and SCODA analysis are plotted against one another. The dots represent metabolites, colored according to their respective classes: amines (pink), bile acids (dark gray), eicosanoids (orange), oxylipins (light blue), free fatty acids (green), lysophospholipids (yellow), and sphingosines (dark blue). The orange dashed lines indicate that metabolites plotted above the line are associated (*p* < 0.05). The red dot-dash lines signify that metabolites above this line are significantly (*q* < 0.05) associated.

**

**

**Figure S4. Progression of SCODA scores in patients with favorable and unfavorable outcomes.** Shown are longitudinal SCODA scores of patients from wave 1 (A, B) and wave 2 (C, D) included in the analysis of associations with progression towards unfavorable outcomes. Panels A and C display patients with an unfavorable outcome; panels B and D show those with a favorable outcome. A favorable outcome was defined as survival from COVID-19 during the study with a final SCODA score ≤ 10; all others were classified as unfavorable. Triangles indicate samples taken during corticosteroid treatment; dots indicate samples without corticosteroid treatment. Line colors denote patient outcomes: red (death), green (discharged alive), blue (hospitalized), and purple (transferred).

**
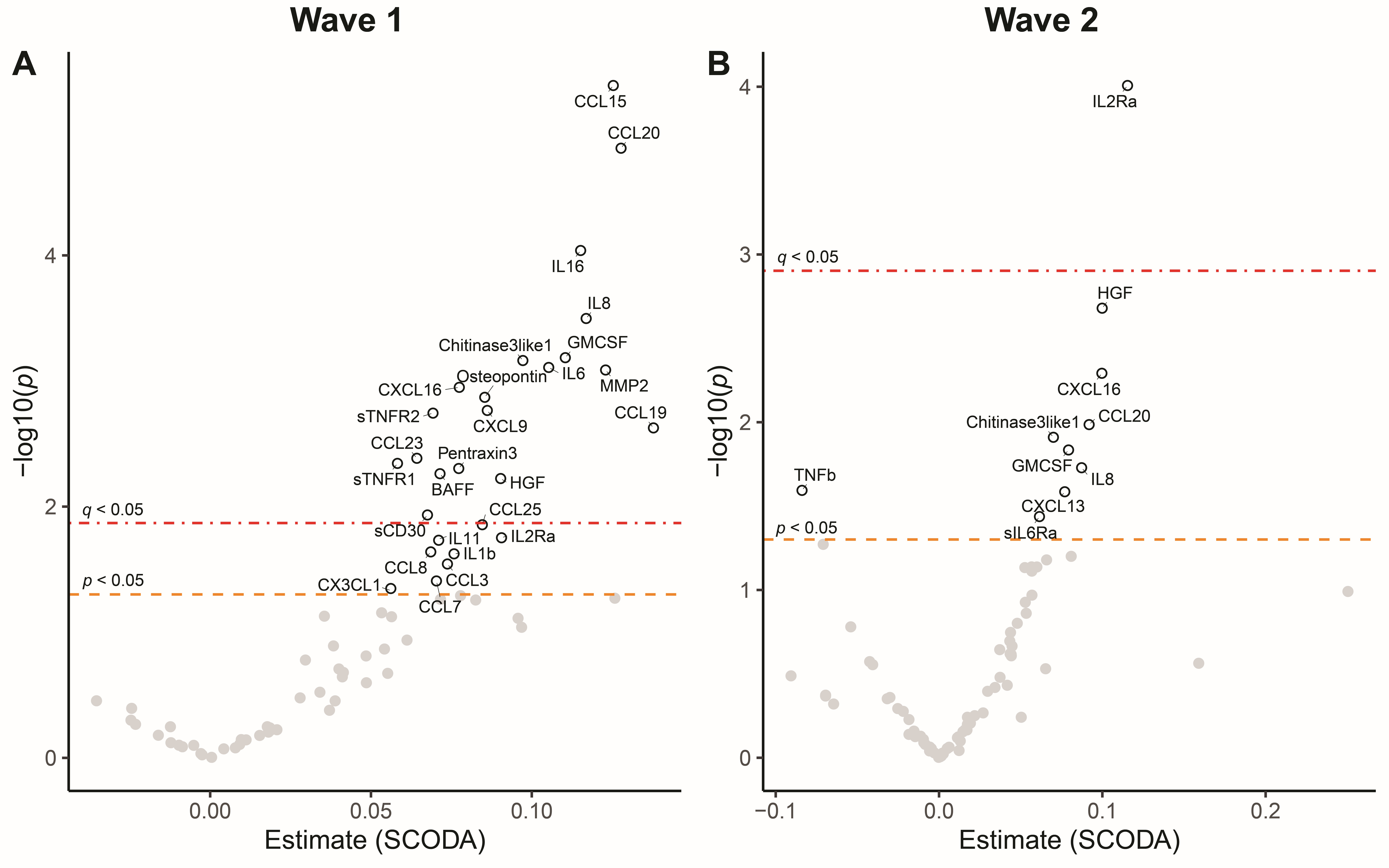
**

**Figure S5. Univariate associations between immune marker level alterations and SCODA score.** Volcano plots show the univariate associations between metabolite levels and SCODA scores for samples from the first wave (A) and second wave (B). Each point represents an immune marker, with the x-axis showing the model estimate and the y-axis the –log10 *p* value. The horizontal orange dashed line marks the threshold for association (*p* < 0.05), while the red dot dash line indicates immune markers significantly associated after multiple testing correction (*q* < 0.05). Positive estimates reflect associations with an increase in SCODA score; negative estimates indicate associations with decreased scores.

**
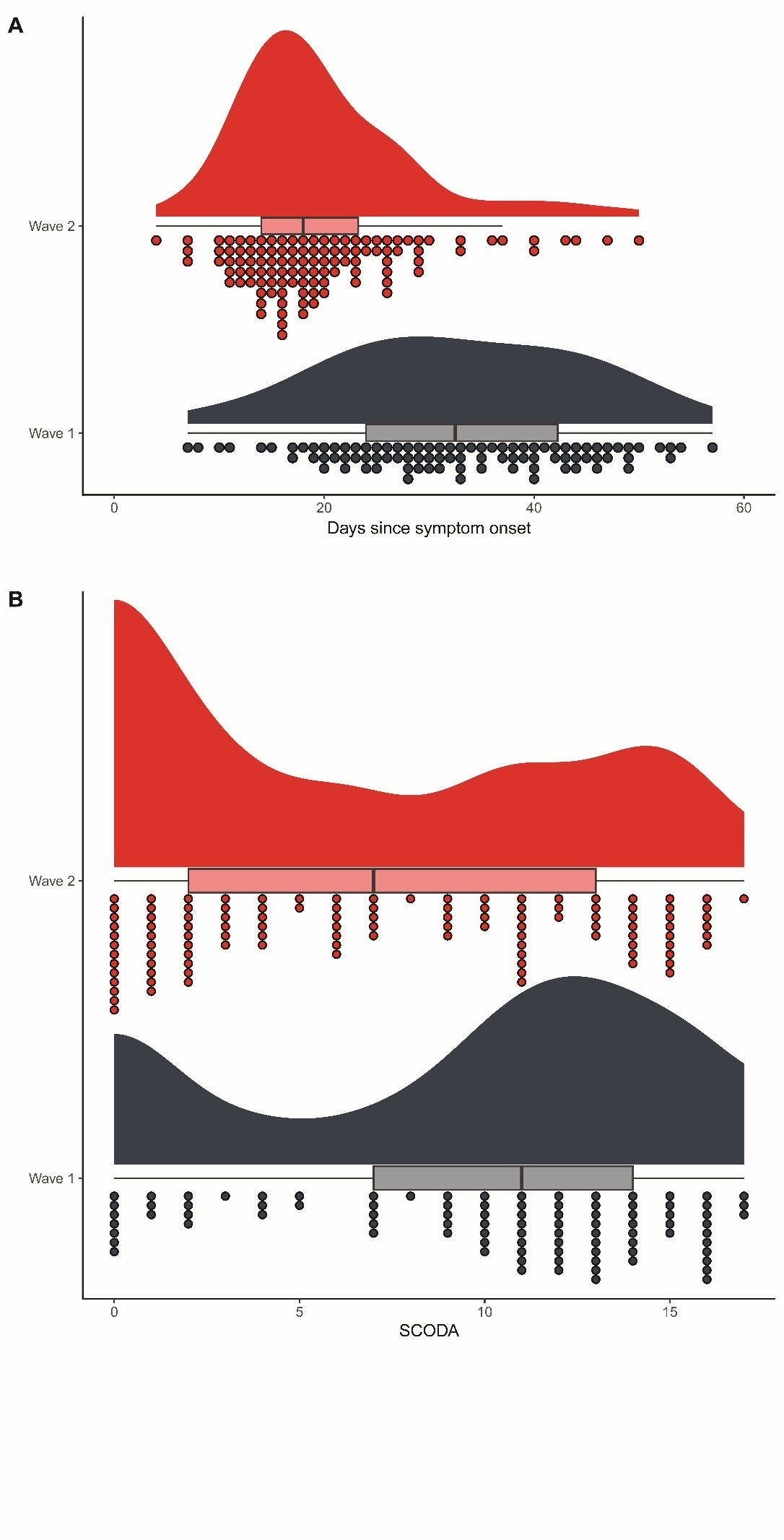
**

**Figure S6. Distribution of samples across the days since symptom onset (A) and SCODA scores (B).**
